# Non-Specific Inhibition of Dipeptidyl Peptidases 8/9 by Dipeptidyl Peptidase 4 Inhibitors Negatively Affects Mesenchymal Stem Cell Differentiation

**DOI:** 10.3390/jcm12144632

**Published:** 2023-07-12

**Authors:** Bárbara Torrecillas-Baena, Marta Camacho-Cardenosa, José Manuel Quesada-Gómez, Paloma Moreno-Moreno, Gabriel Dorado, María Ángeles Gálvez-Moreno, Antonio Casado-Díaz

**Affiliations:** 1 Unidad de Gestión Clínica de Endocrinología y Nutrición-GC17, Instituto Maimónides de Investigación Biomédica de Córdoba (IMIBIC), Hospital Universitario Reina Sofía, 14004 Córdoba, Spain; b42tobab@uco.es (B.T.-B.); marta.camacho@imibic.org (M.C.-C.); md1qugoj@uco.es (J.M.Q.-G.); paloma.moreno.moreno.sspa@juntadeandalucia.es (P.M.-M.); 2 CIBER de Fragilidad y Envejecimiento Saludable (CIBERFES), 14004 Córdoba, Spain; bb1dopeg@uco.es; 3 Departamento Bioquímica y Biología Molecular, Campus Rabanales C6-1-E17, Campus de Excelencia Internacional Agroalimentario (ceiA3), Universidad de Córdoba, 14071 Córdoba, Spain

**Keywords:** DPP4, DPP8/9, vildagliptin, sitagliptin, 1G244, human mesenchymal stem cells, cellular differentiation, osteoblasts, adipocytes

## Abstract

DPP4 may play a relevant role in MSC differentiation into osteoblasts or adipocytes. Dipeptidyl peptidase 4 (DPP4) inhibitors (DPP4i), such as sitagliptin and vildagliptin, are used as antidiabetic drugs. However, vildagliptin is not a specific DPP4i and also inhibits DPP8/9, which is involved in energy metabolism and immune regulation. The aim of this study is to evaluate how sitagliptin, vildagliptin or 1G244 (a DPP8/9 specific inhibitor) may influence cell viability, as well as osteogenic and adipogenic differentiation in human mesenchymal stem cells (MSC). Viability, apoptosis, osteoblastogenesis and adipogenesis markers, as well as protein synthesis of β-catenin, were studied in MSC cultures induced to differentiate into osteoblasts or adipocytes in the presence or absence of sitagliptin, vildagliptin or 1G244. The two tested DPP4i did not affect MSC viability, but 1G244 significantly decreased it in MSC and osteoblast-induced cells. Additionally, 1G244 and vildagliptin inhibited osteogenesis and adipogenesis, unlike sitagliptin. Therefore, inhibition of DPP4 did not affect MSC viability and differentiation, whereas inhibition of DPP8/9 negatively affected MSC. To the best of our knowledge, these results show for the first time that DPP8/9 have an important role in the viability and differentiation of human MSC. This data can be considered for human clinical use of drugs affecting DPP8/9 activity.

## 1. Introduction

Dipeptidyl peptidase 4 (DPP4) or cluster of differentiation 26 (CD26; E.C. 3.4.14.5) is a protease ubiquitously synthesized. It has been detected in numerous organs and tissues such as the lung, kidney, pancreas, uterus, prostate, blood vessels, brain, thymus, bone, lymph nodes and spleen. It is found on the surface of different cells, including epithelial, endothelial and immune ones, among others. In addition, transmembrane-domain cleavage releases a soluble form of the enzyme that can be identified in blood plasma [1]. Through its protease activity, it can cleave various substrates such as chemokines, growth factors, fibronectin, neuropeptides like neuropeptide Y (NPY) and substance P, as well as incretin hormones [gastric inhibitory polypeptide or glucose-dependent insulinotropic polypeptide (GIP) and glucagon-like peptide-1 (GLP-1)], thereby regulating their concentrations and functions. Due to the diversity of substrates on which it acts, its activity plays a role in regulating numerous cellular processes such as proliferation, adhesion, differentiation, immunomodulation and apoptosis [2,3].

Among the main substrates of DPP4 are GLP-1 and GIP incretins. These hormones are secreted by gut L and K cells, respectively. Their physiological functions involve control of blood glucose concentration. This is accomplished by stimulating insulin secretion or decreasing glucagon production [4]. Circulating incretins are rapidly degraded by DPP4. Therefore, inhibition of this protease increases GLP1 levels and, thus, the ability to metabolize glucose. On this basis, numerous DPP4 inhibitor (DPP4i) molecules have been developed as antidiabetic drugs. These molecules belong to the gliptin family. Among others, they include sitagliptin, saxagliptin, linagliptin and vildagliptin. DPP4i increases GLP-1 and GIP levels, which inhibit glucagon release, increase insulin secretion and decrease gastric emptying, as well as blood glucose levels in diabetic patients [5]. The advantages of these drugs include that they have a very low risk of hypoglycemia, are very well tolerated and have a neutral effect on weight [6].

DPP4 is able to recognize different substrates involved in different physiological functions. Therefore, the generalized use of DPP4i for type-2 diabetes mellitus (T2DM) treatment has revealed other effects derived from DPP4 inhibition [7]. Thus, in recent years, the study of how DPP4 inhibition can affect other pathologies associated with cardiovascular or renal systems, as well as bone metabolism, for example, has increased; many of them are related to T2DM [8,9,10].

DPP4 activity modulates the physiology of different cell types and tissues. Among them are mesenchymal stem cells (MSC) [11,12,13]. MSC in adults are involved in organism homeostasis. They mainly participate in tissue regeneration under both physiological and pathological conditions [14]. MSC have been detected in different tissues, including bone marrow, fatty ones, periodontal ligament, synovium, umbilical cord, placenta and hair follicle, among others [15]. In vitro, they are characterized by their high proliferation capacity, adherence to plastic and ability to differentiate into osteoblasts, adipocytes or chondrocytes under different stimuli. In addition to their differentiation ability, the presence of surface markers, such as CD73, CD90 and CD105, as well as the absence of haematopoietic markers, like CD34 and CD45, have been used to define them [16]. They can be manipulated and cultured in vitro, exhibiting interesting immunomodulatory and differentiation capacities. Therefore, MSC are a tool with high potential for applications in regenerative medicine [17].

In bone marrow, during aging, MSC may differentiate more into adipocytes than osteoblasts. This increases adiposity in bone marrow, loss of bone mass and risk of fractures [18]. Some studies have associated high plasma DPP4 levels with increased bone turnover, as well as increased prevalence of osteoporotic fractures in postmenopausal women [19]. Furthermore, it has been found that in normoglycemic postmenopausal women, DPP4 activity is associated with an increased risk of osteoporosis, insulin resistance, inflammation and decreased GLP-1 levels [20]. In addition, high plasma DPP4 levels in type II diabetic patients have been linked with bone loss, as well as an increased risk of bone fractures [21].

Among the DPP4i used for the treatment of T2DM, there are some that are not specific for DPP4 and can also inhibit DPP8/9 [22]. Therefore, these DPP4i may have additional effects on patients besides their antidiabetic effects. DPP8/9, as DPP4, belong to the dipeptidyl peptidase 4 activity and/or structure homologues (DASH) family [23]. But unlike DPP4, they do not possess a transmembrane domain. Therefore, their localization is exclusively cytoplasmic [24]. The expression of genes encoding DPP8/9 in the organism is ubiquitous. They have been related to cell behavior, energy metabolism and immune regulation [25]. However, their putative roles in the viability and differentiation of MSC have not been studied so far. Therefore, the aim of this study is to evaluate how DPP4 and/or DPP8/9 inhibition may influence the viability and osteogenic/adipogenic differentiation of MSC. Such knowledge should shed new light on the development of new therapeutic strategies in regenerative medicine for the treatment of pathologies including T2DM, osteoporosis and obesity. It will also provide information on possible side effects of certain non-specific DPP4i drugs on patients with T2DM, allowing them to take appropriate precautions, if necessary. To achieve these goals, human bone marrow MSC were treated with 1G244 (a specific DPP8/9 inhibitor) or DPP4i drugs (vildagliptin and sitagliptin). The former can inhibit DPP8/9, in addition to DPP4, whereas the latter is specific for DPP4 [22].

## 2. Materials and Methods

### 2.1. MSC Culture and Differentiation

Human bone marrow MSC were obtained from cryopreserved and previously characterized cultures belonging to our cell collection [26] (See Supplementary Material Figures S1 and S2). Cells were thawed and seeded in 75 cm^2^ flasks from Nalgene-Nunc-Thermo Fisher Scientific (Waltham, MA, USA). They were grown in Alpha Minimum Essential Medium (α-MEM) from Cambrex Bio Science–Lonza (Basel, Switzerland), containing 2 mM UltraGlutamine (Lonza), 10% fetal bovine serum (FBS) (Gibco-Thermo Fisher Scientific), 100 U ampicillin, 0.1 mg streptomycin/mL and 1 ng basic fibroblast-growth factor (bFGF)/mL from Sigma-Aldrich (Saint Louis, MO, USA). Cultures were incubated at 37 °C, with 5% CO_2_ and 95% humidity. Culture media were changed every 3 or 4 days.

Cells were detached with trypsin-EDTA (Gibco) when cultures reached near 90% confluence. After 3 or 4 passages, MSC were seeded in culture plates (Nalgene-Nunc-Thermo Fisher Scientific) at a density of about 1000 cells/cm^2^. Once a confluence between 60 and 80% was reached, they were induced to differentiate into adipocytes or osteoblasts. To induce adipocyte differentiation, culture media were supplemented with 5 × 10^−7^ M dexamethasone, 50 μM indomethacin and 0.5 mM isobutylmethylxanthine. During differentiation, two stages were considered for collecting samples (preadipocytes at 6 to 7 days and mature adipocytes at 13 to 14 days). On the other hand, differentiation into osteoblasts was carried out by supplementing culture media with 10^−8^ M dexamethasone, 10 mM β-glycerolphosphate and 0.2 mM ascorbic acid. Cultures induced into osteoblasts were grown for three to four weeks to allow extracellular mineralization. All inducers were from Sigma-Aldrich.

### 2.2. Vildagliptin, Sitagliptin or 1G244 Treatments

MSC cultures not differentiated or induced to differentiate into adipocytes or osteoblasts were treated with 10 μM of vildagliptin from Selleckchem (Houston, TX, USA), sitagliptin or 1G244 (both from Sigma-Aldrich). Cultures maintained under the same conditions but not treated with inhibitors were used as controls.

### 2.3. Dipeptidyl Peptidase Activity

Dipeptidyl peptidase activity was measured in culture media of different treatments, according to the following protocol: 30 μL of culture medium plus 100 μL of reaction mixture 0.5 mM of Gly-Pro 4-Methoxy-Beta-Naphthylamide (H-Gly-Pro-4MβNA) dipeptidyl peptidase-specific substrate for DPP4 or DPP8/9 [27], from Bachem (Bubendorf, Switzerland) in 50 mM Tris-HCl pH 8 (Sigma-Aldrich) were mixed and incubated at 37 °C for 20 min. Then, fluorescence at 360 nm excitation and 450 nm emission was quantified in an Infinite F200 Pro fluorometer from Tecan (Mannedorf, Switzerland). Controls used were α-MEM without FBS, as negative, and α-MEM 10% FBS, as positive.

### 2.4. MTT Assay for Cell Viability

Cell viability was determined using 3-(4,5-dimethylthiazolyl-2)-2,5-diphenyltetrazolium bromide (MTT) (Sigma-Aldrich). MSC were seeded in 96-well plates at a density of 4000 cells per well in culture media. They were incubated for 24 h prior to treatment. Subsequently, cells were treated with 10 μM of vildagliptin, sitagliptin or 1G244. After 48 h, media were removed and 100 µL of MEMα supplemented with 1 mg MTT/mL was added. After 2 h of incubation in culture conditions, solutions were removed. Insoluble formazan crystals produced were dissolved in isopropanol. Absorbance at 570 nm of resulting solutions was measured, using absorbance at 650 nm as reference, with a PowerWave XS microplate spectrophotometer from BioTek Instruments (Winooski, VT, USA). In MSC cultures induced to differentiate into adipocytes and osteoblasts in presence of vildagliptin, sitagliptin or 1G244, cell viability was measured on day seven after differentiation started, as described above.

### 2.5. Apoptosis Assay

Culture media were removed, and apoptotic cells were detected with 4 µM Caspase 3/7 reagent (CellEvent) from Thermo Fisher Scientific in phosphate-buffered saline (PBS) with 5% FBS. After incubation for 1 h at 37 °C, cells were fixed with 3.7% formaldehyde, and nuclei were stained with Hoechst stain from Sigma-Aldrich. Images were taken with a fluorescence microscope and analyzed with Image J software version from National Institutes of Health (NIH; Bethesda, MD, USA) <https://imagej.nih.gov/ij>. Caspase-activity signals and the corresponding Hoechst-staining signals were quantified. Apoptosis was calculated as caspase/Hoechst staining ratios.

### 2.6. Mineralized Extracellular-Matrix (Osteoblasts) and Lipid Droplet (Adipocytes) Staining

Mineralization in cultures induced to differentiate into osteoblasts was evaluated by staining with alizarin red S. For this purpose, cultures were fixed with 3.7% formaldehyde for 10 min at room temperature. Then, they were stained for 10 min with a 40 mM solution of alizarin red S (pH 4.1) from PanReac AppliChem (Darmstadt, Germany) and then washed with 60% isopropanol. For quantification, stainings were eluted with 10% acetic acid and neutralized with 10% ammonium hydroxide. Absorbance of the resulting solutions were measured at 405 nm using a PowerWave XS microplate spectrophotometer.

Formation of lipid droplets in cultures induced to differentiate into adipocytes was evaluated by oil red O staining. Cultures were fixed with 3.7% formaldehyde for 15 min and stained with a solution prepared by mixing six volumes of oil red O at 0.35% (*w*/*v*, in isopropanol) with four volumes of distilled water. After 20 min of incubation, cells were washed with distilled water and stained with hematoxylin. Optical microscopy pictures were then taken. For quantification of stainings, oil red O was eluted with 100% isopropanol for 10 min at room temperature, and absorbance was measured at 510 nm by spectrophotometry (PowerWave XS). Values of oil red O stain were normalized, considering the number of cells per well, as estimated by crystal-violet staining. Stains were eluted with 10% acetic acid for 20 min, and quantification was performed at 590 nm by spectrophotometry (PowerWave XS). Lipid-droplet formation in cell cultures was calculated as A_510_ nm/A_590_ nm. Additionally, images obtained by optical microscopy were analyzed by Image J software 1.53f51. Thus, formation of lipid droplets was quantified by both methods.

### 2.7. Quantification of Gene Expression by Quantitative Real-Time PCR

RNA was isolated using NZY total RNA isolation kit from NZYTech (Lisbon, Portugal), following the manufacturer’s instructions. Nucleic acids were quantified with a NanoDrop ND-1000 Spectrophotometer from Thermo Fisher Scientific. Next, 900 ng of RNA were retrotranscribed into cDNA, using iScript cDNA Synthesis Kit from Bio-Rad (Hercules, CA, USA), according to the manufacturer’s directions.

Quantitative real-time PCR (qRT-PCR) was carried out in a LightCycler 96 Instrument from Roche Applied Science (Penzberg, Germany). Each PCR reaction was performed in a 10 µL volume containing 1 µL of cDNA, 10 pmol of each primer pair (Table 1) and 1X of SensiFAST Sybr No-Rox Mix from Bioline (London, UK). The PCR amplification program included one cycle at 95 °C for 2 min (DNA denaturation) and 40–45 cycles of 95 °C for 5 s (DNA denaturation) and 65 °C for 30 s (primer hybridization and extension by DNA polymerase). Results were analyzed with LightCycler 1.1 software from the same manufacturer. Polymerase (RNA; Targeted DNA) II polypeptide A (POLR2A) was used as housekeeping gene. Relative gene expression respect control (value = 1) was calculated using the 2^−ΔΔCt^ method, where Ct is the cycle threshold.

### 2.8. Western Blot

Cells were lysed with Cell Extraction Buffer (Thermo Fisher Scientific) supplemented with 1 mM of phenylmethylsulfonyl fluoride (PMSF) and 50 μL of protease inhibitor cocktail (PIC)/mL (both from Sigma-Aldrich) for total protein isolation. Lysates, once collected, were incubated in ice for 30 min, with vortex agitation every 10 min. Finally, they were centrifuged for 10 min (13,000× *g*) at 4 °C. Supernatants were transferred into new tubes and stored at −20 °C until used. Protein concentrations were quantified with the DC Protein Assay kit (Bio-Rad) according to the manufacturer’s directions.

Subsequently, 15–20 μg of protein from each sample were loaded into 8–16% acrylamide nUView Tris-Glycine Precast Gels from NuSeP (Germantown, MD, USA) under denaturing conditions. Electrophoreses were carried out in a Mini-Protean (Bio-Rad) system. Then, proteins were transferred into polyvinylidene difluoride (PVDF) membranes (Bio-Rad) by a Trans-Blot Turbo Transfer System from the same manufacturer. Membranes were blocked with a 5% solution of skimmed milk in Tris–Tween-Buffered Saline (TTBS) buffer (20 mM Tris-HCl pH 7.6, 150 mM NaCl, 0.05% Tween) for one hour at room temperature. Subsequently, membranes were incubated overnight at 4 °C, with primary antibody anti-β-catenin (1:1000) from Cell Signaling Technology (Danvers, MA, USA), in 1% skimmed milk in TTBS. After incubation, membranes were washed with TTBS and incubated with the secondary anti-Rabbit IgG H&L-HRP antibody (1:3000) from Abcam (Cambridge, UK) in 1% skimmed milk in TTBS for one hour. Finally, membranes were developed with Clarity Western ECL Substrate (Bio-Rad). Stain-free technology was used with nUView Tris-Glycine Precast Gels. In short, UV light was used to activate samples in the ChemiDoc XRS+ Gel Imaging System (Bio-Rad). Images of total protein loaded for each sample were generated. They were analyzed with Image Lab software version 6.0 from the same company. Total protein signals were quantified [28,29]. Values obtained were used for normalization of band intensity of β-catenin.

### 2.9. Statistical Analyses

GraphPad Prism 6.0 program from GraphPad Software (San Diego, CA, USA) was used to analyze data and generate plots. Comparison of different treatments was performed using analysis of variance (ANOVA) tests to detect significant changes. This was followed by a Tukey’s test to identify significant differences between pairs of treatments. Differences were considered statistically significant when *p* < 0.05. At least three data per parameter studied were obtained. All graphs show mean plus standard error of the mean (mean ± SEM).

## 3. Results

### 3.1. Dipeptidyl Peptidase Activity in Culture Media

**Figure 1:**
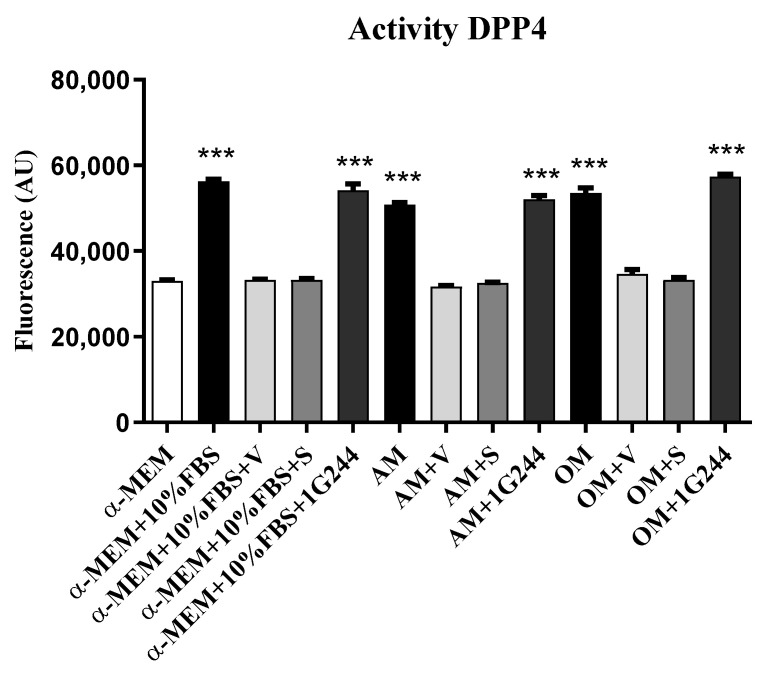
Dipeptidyl peptidase activities in culture media. Measurements were performed in different culture media, including the following: (i) α-MEM; (ii) α-MEM supplemented with 10% FBS; and (iii) α-MEM supplemented with 10% FBS and inducers to differentiate MSC into adipocytes (AM) or osteoblasts (OM), in presence or absence of vildagliptin (V), sitagliptin (S) or 1G244. AU: arbitrary units. *** *p* < 0.001 vs. controls (untreated).

DPP4i in the treatment of T2DM are mainly directed to the inhibition of soluble DPP4. As the aim of this study was to understand how DPP4i can affect MSC and their differentiation, we first determined whether the culture medium where the cells were grown and differentiated contained soluble DPP4 activity. Unsupplemented culture medium (α-MEM) consists of a well-defined solution of salts, vitamins, amino acids and sugars, with no proteins present. The possible source of DPP4 activity in the medium was FBS, with which it was supplemented. Therefore, dipeptidyl peptidase activity in α-MEM + 10% FBS medium supplemented or not supplemented with the differentiation inducers was first studied. As expected, α-MEM alone did not have dipeptidyl peptidase activity. However, the medium supplemented with FBS had this activity, which was inhibited in the presence of 10 μM vildagliptin or sitagliptin, indicating the presence of DPP4 activity in the culture medium. Furthermore, the use of 1G244 did not inhibit dipeptidyl peptidase activity, as expected, since there was no soluble DPP8/9 in the serum. The presence of inducers in the osteogenic (OM) or adipogenic (AM) medium did not modify results (Figure 1). Therefore, culture media (α-MEM + 10% FBS), in which the different experiments were performed, represent the basal DPP4 conditions in which cells were grown.

### 3.2. Effect of DPP4 and DPP8/9 Inhibition on MCS Viability and Apoptosis

**Figure 2:**
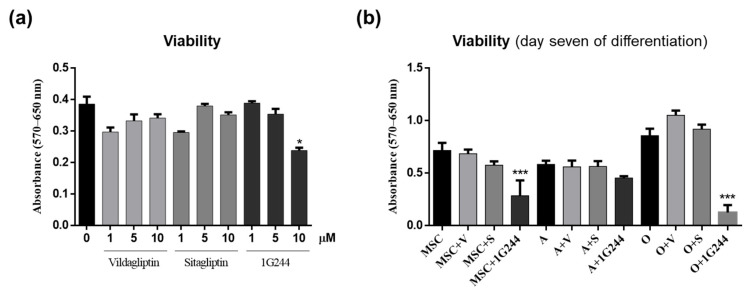
Effects of vildagliptin, sitagliptin or 1G244 on cell viability. (**a**) Quantification was performed on cultures of MSC treated with different concentrations of these chemicals for 48 h. (**b**) Viability quantification in cultures of MSC not differentiated or induced to differentiate into adipocytes (A) or osteoblasts (O) for seven days, non-treated or treated with 10 μM of vildagliptin, sitagliptin or 1G244. V: vildagliptin; S: sitagliptin. * *p* < 0.05; *** *p* < 0.001 vs. control (untreated).

In order to find out whether inhibition of DPP4 or DPP8/9 could affect the viability of MSC cultures, the effect of different doses of the inhibitors on undifferentiated MSC cultures were evaluated. Thus, cells were treated with 0, 1, 5 and 10 μM of vildagliptin, sitagliptin or 1G244 for 48 h. Then, cell viability was quantified by MTT assays. Results showed that viability decreased by about 35% in cultures treated with 10 μM 1G244. Yet, those treated with different concentrations of vildagliptin or sitagliptin showed no significant changes in cell viability compared to controls (Figure 2a).

Taking into account these results, a concentration of 10 µM of different inhibitors was chosen for subsequent studies. The reasoning was that vildagliptin, DPP4i and DPP8/9 partial inhibitors did not affect viability at such concentration. Therefore, it was of interest to check if it could affect MSC differentiation with respect to sitagliptin (specific DPP4i). To this end, we first studied the effects of treatments with vildagliptin, sitagliptin or 1G244 on the viability of MSC cultures (differentiated or not into adipocytes or osteoblasts for seven days) in the presence or absence of DPP4 or DPP8/9 inhibitors. Results showed a significant decrease in viability of not differentiated MSC and osteoblast-induced cultures treated with 1G244, mainly for the latter ones. Conversely, those treated with vildagliptin and sitagliptin showed no changes in cell viability. It is interesting to note that, in MSC cultures induced to differentiate into adipocytes, none of the treatments affected cell viability. However, a decreasing trend was observed in cultures treated with 1G244 (Figure 2b).

**Figure 3:**
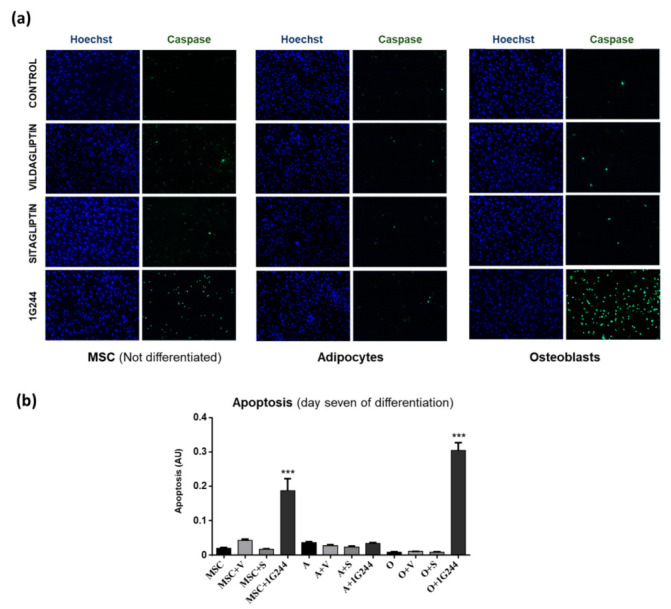
Effects of vildagliptin, sitagliptin or 1G244 on apoptosis. (**a**) Representative images of caspase activation and Hoechst nuclei staining in cultures of MSC, not differentiated or differentiated into osteoblasts or adipocytes, treated with 10 μM of vildagliptin, sitagliptin or 1G244 for seven days. (**b**) Apoptosis quantification of treatments described in (**a**). AU: arbitrary units of caspase staining/Hoechst staining. *** *p* < 0.001 vs. control (untreated).

In addition, taking into account the above results, apoptosis was also measured in undifferentiated or induced-to-differentiate MSC cultures for seven days, treated as described above. Results were similar to those obtained for viability. Thus, apoptosis increased only with 1G244 treatment in undifferentiated and osteoblast-differentiated MSC, whereas in adipocytes, no significant changes were seen with any of the treatments (Figure 3). Taken together, the viability and apoptosis results showed that the role of DPP8/9 in adipocytes was different from the one in MSC and osteoblasts.

### 3.3. Effect of DPP4 and DPP8/9 Inhibition on Osteoblastic Differentiation

**Figure 4:**
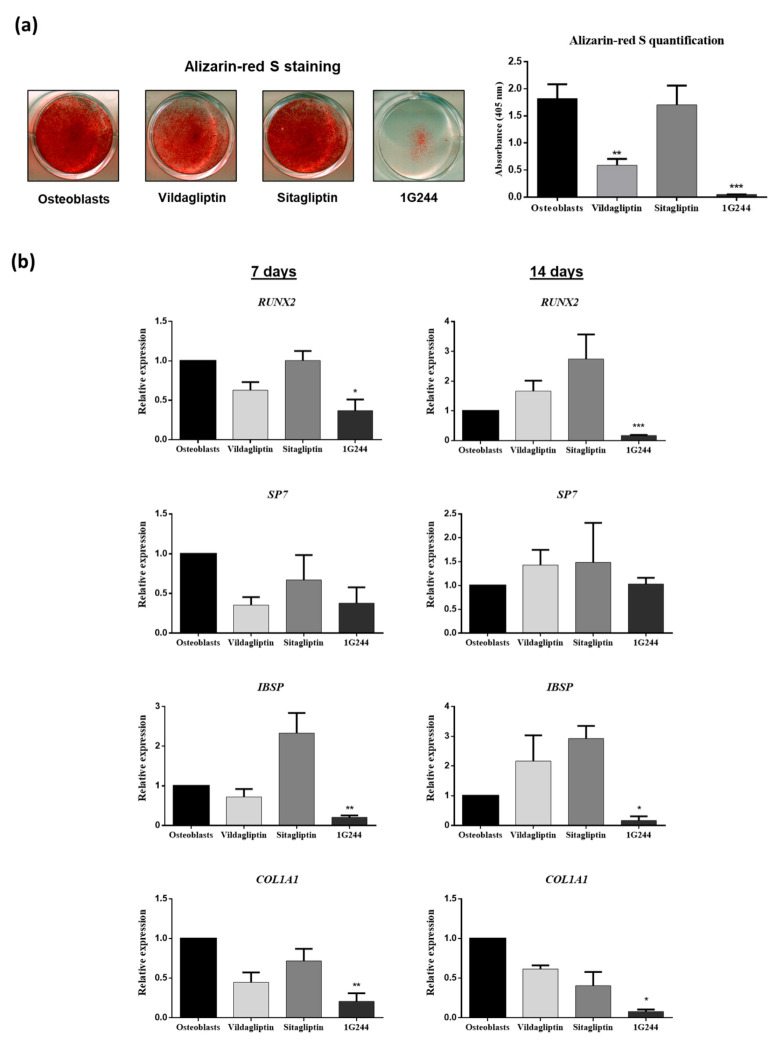
Effects of vildagliptin, sitagliptin or 1G244 in osteoblastic differentiation. Cultures of MSC were induced to differentiate into osteoblasts. (**a**) Osteoblast mineralization. Representative images of cultures stained with alizarin red S, on day 21 of osteoblastic differentiation, in cultures treated with these chemicals. The graph represents dye elution quantification by spectrophotometry. (**b**) Gene expression of osteoblastic marker genes (RUNX2, SP7, IBSP and COL1A1) on days 7 and 14 after onset of osteoblastic differentiation, in presence or absence of vildagliptin, sitagliptin or 1G244 chemicals. * *p* < 0.05; ** *p* < 0.01; *** *p* < 0.001 vs. control (untreated).

To evaluate whether inhibition of DPP4 or DPP8/9 could affect osteogenic differentiation of MSC, cultures induced to differentiate into osteoblasts were treated with 10 μM vildagliptin, sitagliptin or 1G244 during the differentiation process. After 21 days of differentiation and treatment, significant decreases in extracellular-matrix mineralization were observed in cultures treated with vildagliptin and mainly with 1G244, as shown by alizarin red S staining (Figure 4a). Expressions of osteoblastic gene markers in these cultures were studied on days 7 and 14 after the initiation of differentiation. Thus, the gene encoding runt-related transcription factor 2 (RUNX2) was downregulated on days 7 and 14, with vildagliptin or 1G244 being statistically significant in cultures treated with 1G244 (Figure 4b). However, treatments did not produce significant changes in the expression of the gene encoding osterix (SP7) transcription factor. Nevertheless, a tendency to decrease was observed with vildagliptin or 1G244 on day seven (Figure 4b). Expressions of integrin-binding sialoprotein (IBSP) and collagen, type I, alpha 1 (COL1A1) genes, encoding extracellular matrix proteins, were also repressed in cultures treated with 1G244. No significant changes were observed in cultures treated with the two evaluated DPP4i. Although, in the case of IBSP, treatment with sitagliptin tended to increase its expression, mainly on day seven of differentiation (Figure 4b). Thus, these results indicate that inhibition of DPP8/9 by 1G244 downregulated osteoblastic-marker gene expression, further preventing mineralization in osteoblastogenesis.

**Figure 5:**
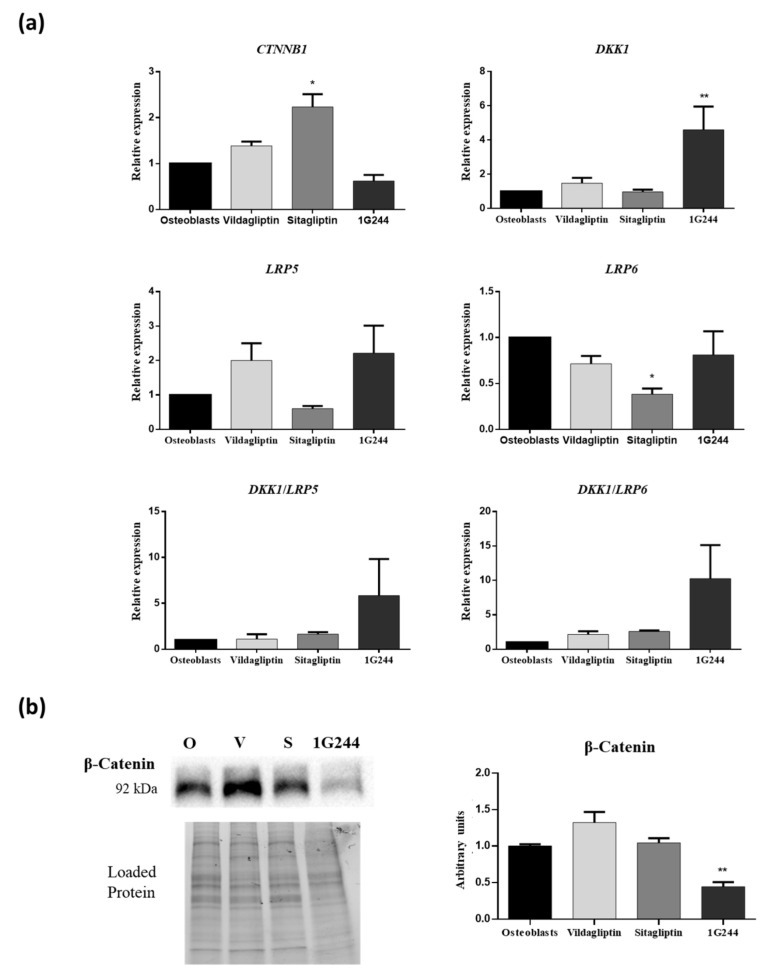
Effects of vildagliptin, sitagliptin or 1G244 in β-catenin, DKK1, LRP5 and LRP6 gene expression in MSC cultures induced to differentiate into osteoblasts. (**a**) Expression of *CTNNB1*, *DKK1*, *LRP5* and *LRP6* genes on day 14 of osteogenic differentiation. (**b**) Western blot for β-catenin protein of cultures treated as indicated before. Results of the quantification of Western blot bands are shown. O: osteoblasts; V: vildagliptin; S: sitagliptin. * *p* < 0.05; ** *p* < 0.01; vs. control (untreated).

Interestingly, the β-catenin pathway plays an important role in osteogenic differentiation. Thus, it was analyzed if inhibition of DPP4 or DPP8/9 could affect it during osteogenesis. Therefore, expression of the gene encoding β-catenin (*CTNNB1*), as well as the ones encoding dickkopf Wnt signaling pathway inhibitor 1 (*DKK1*) and low-density lipoprotein receptor-related protein 5 or 6 (*LRP5/6*), involved in inhibition or activation of the β-catenin pathway were studied. This was carried out during osteoblastic differentiation, in the presence of vildagliptin, sitagliptin or 1G244, on day 14. Results showed that sitagliptin treatment increased *CTNNB1* gene expression. However, inhibition of DPP8/9 by 1G244 significantly increased expression of the gene coding DKK1, which is an inhibitor of the β-catenin pathway (Figure 5a). On the other hand, *LRP5* and *LRP6* genes encode proteins that activate the β-catenin pathway. Thus, the relationship between *DKK1* expression and one of the other genes is indicative of possible activation or inhibition of the β-catenin pathway by the different treatments used. *DKK1*/*LRP5* and *DKK1*/*LRP6* gene expression ratios are shown in Figure 5a. Results showed an increase of both with 1G244 treatments, although they were not statistically significant. These results are in line with those obtained by analyzing β-catenin protein synthesis by Western blot They showed that 1G244 decreased the synthesis of this protein on day 14 of osteoblastic differentiation (Figure 5b).

### 3.4. Effect of Vildagliptin, Sitagliptin or 1G244 on Adipogenic Differentiation

**Figure 6:**
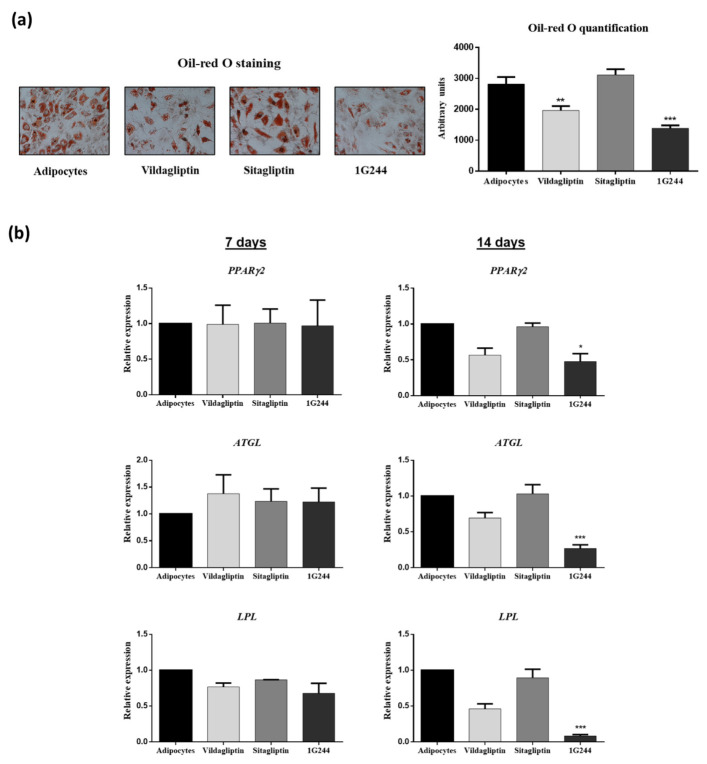
Effects of vildagliptin, sitagliptin or 1G244 on adipogenic differentiation. MSC cultures were induced to differentiate into adipocytes. (**a**) Representative light-microscopy pictures (200X) of oil red O staining of lipid droplets on day 14 of adipocyte differentiation in presence of 10 μM of these chemicals. Results of spectrophotometric quantification of dye elutions are shown in the plot. (**b**) Gene expression of adipocyte marker genes (*PPARG2*, *ATGL* and *LPL*) on days 7 and 14 after starting adipocyte differentiation. * *p* < 0.05; ** *p* < 0.01; *** *p* < 0.001 vs. control (untreated).

To study whether DPP4 or DPP8/9 inhibition could affect MSC adipogenesis, cultures of these cells were differentiated into adipocytes in the presence or absence of 10 μM of vildagliptin, sitagliptin or 1G244. Results obtained showed that treatment with 1G244, followed by vildagliptin during 14 days of adipogenic differentiation, significantly decreased lipid-droplet formation. This was made evident by oil red O staining quantification (Figure 6a). Interestingly, expressions of marker genes such as peroxisome proliferator-activated receptor gamma 2 (*PPARG2*; encoding a master transcription factor in adipocyte differentiation), adipose triglyceride lipase (*ATGL*) and lipoprotein lipase (*LPL*) on day seven were not significantly affected by the presence of vildagliptin, sitagliptin or 1G244. However, the down-regulation of these genes involved in fatty-acid metabolism was mainly observed on day 14, with 1G244, followed by vildagliptin treatments. Decrease in mRNA levels were statistically significant for cultures treated with 1G244 (Figure 6b).

**Figure 7:**
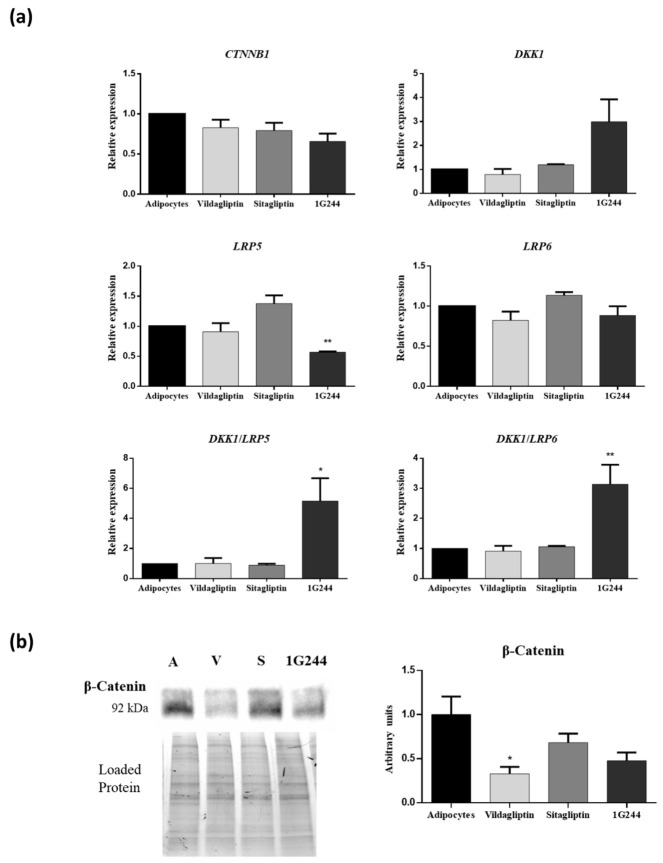
Effects of vildagliptin, sitagliptin or 1G244 in β-catenin, DKK1, LRP5 and LRP6 expression in MSC cultures induced to differentiate into adipocytes. (**a**) Gene expression of CTNNB1, DKK1, LRP5 and LRP6 genes on day 14 of osteogenic differentiation. (**b**) Western blot for β-catenin protein of cultures treated with these chemicals on day 14 of adipogenic differentiation. Results of the quantification of Western blot bands are shown. A: adipocytes; V: vildagliptin; S: sitagliptin. * *p* < 0.05; ** *p* < 0.01 vs. control (untreated).

On the other hand, because the β-catenin pathway also influences adipogenic differentiation of MSC, the expression of genes involved in this pathway and β-catenin synthesis were studied. Expression of *CTNNB1, DKK1* and *LRP6* genes did not show significant changes with any of the treatments. However, cultures treated with 1G244 significantly repressed *LRP5* and induced *DKK1/LRP5* and *Dkk1/LRP6* gene expression ratios (Figure 7a). This suggests a possible inhibition of the β-catenin pathway. Indeed, quantification of β-catenin protein levels showed that on day 14 after adipogenic induction, cultures treated with vildagliptin or 1G244 had the lowest values (Figure 7b).

## 4. Discussion

During aging and in certain pathologies, bone marrow adipocytes inhibit hematopoietic and bone regeneration. This is accomplished, in part, through the secretion of large amounts of DPP4 [30,31]. Indeed, such secretion takes place in bone marrow niches. This protease recognizes diverse substrates with important physiological functions [32]. In fact, its activity may affect the differentiation of osteoblast- and adipocyte-precursor cells. On the other hand, the use of DPP4i is generalized as antidiabetic. Thus, it is important to understand whether the DPP4 inhibition could affect another physiological process besides glucose metabolism. Among them are numerous ones related to MSC biology, as recently reviewed by our group [11]. Therefore, knowledge of how inhibition of DPP4 (or other DPP, as DPP8/9 affected by non-specific DPP4i) may influence MSC viability and differentiation is important. For instance, this kind of knowledge would be invaluable for developing MSC-based therapeutic strategies targeting T2DM patients consuming these drugs and, likewise, for the identification of new potential applications of DPP4i related to regenerative medicine. Our results indicate that inhibition of DPP4 by sitagliptin did not significantly affect MSC viability and differentiation. Yet, inhibition of DPP8/9 by vildagliptin, and mainly by 1G244, decreased viability and inhibited osteogenic and adipogenic differentiation of MSC. This suggests that DPP8/9 play key roles in maintaining the viability and differentiation capacity of MSC.

Indeed, DPP8/9 have been implicated in different biological processes related to energy metabolism, inflammation, and cell behavior. The functions of these proteins can be independent of their enzymatic activity or be mediated by their peptidase activity. Thus, in the latter case, inflammatory proteins, chemokines and growth factors have been identified among their natural substrates [25]. To the best of our knowledge, our data show for the first time that DPP8/9 are also involved in the maintenance of human MSC viability, as well as in their differentiation into osteoblasts and adipocytes.

Cell viability decreased when DPP8/9 activity was inhibited with 10 µM of 1G244 specific inhibitor, but not with the same concentration as vildagliptin. The latter inhibited DPP4, but with respect to 1G244, it had less power to inhibit DPP8/9. This showed that DPP4 inhibition did not affect viability, as was also shown by treatment with sitagliptin as a specific DPP4 inhibitor. These results suggest that DPP8/9 activity is important for the maintenance of cell viability in MSC. This may explain the in vivo effects in preclinical models identified by other authors. For instance, they showed that different doses of a specific DPP8/9 inhibitor produced alopecia, thrombocytopenia, reticulocytopenia, enlarged spleen, multiorgan histopathological changes and mortality in rats. Additionally, they produced gastrointestinal toxicity in dogs. However, DPP4 inhibition had no cytotoxic effects in these species [33]. Yet, there is controversy about whether the effects observed with the specific DPP8/9 inhibitors were due to inhibition of the activity of these peptidases or to some cytotoxic effects of the inhibitor used. Another study with vildagliptin showed that maintaining high plasma doses (that should inhibit DPP8/9) did not generate toxic effects in mice and rats. They concluded that loss of DPP8/9 activity had no adverse effects [34]. Others denied the in vivo toxicity of 1G244. Nevertheless, the possible contribution of some undesirable effects of high concentrations of this DPP8/9 inhibitor could not be excluded [35]. These studies were only carried out for 14 days. However, Longer periods might produce adverse effects due to possible deterioration of progenitor-cell populations.

On the other hand, it has been shown that loss of DPP9 activity resulted in neonatal death 24 h after birth in dipeptidyl peptidase 9 (DPP9) knock-in mice. This was due to an increase in apoptosis of occipital somite-derived migratory tongue-muscle progenitors. This caused the loss of the sucking ability of those mice. This effect was specific to DPP9 activity. Furthermore, DPP8 activity in those cells was not able to compensate for the absence of DPP9 [36]. This study supports our results on the importance of DPP9 activity in maintaining progenitor-cell viability, like MSC.

Moreover, it has been shown that 10 µM of 1G244 in skin-cell cultures of fibroblasts and human epidermal keratinocyte (HaCaT) cells decreased proliferation in vitro [37]. In monocytes, DPP9 activity has also been shown to be important for viability and differentiation into macrophages. Thus, DPP9 increased during differentiation into M1 and M2 macrophages. Furthermore, treatment with 1G244 inhibited M1 macrophage activation and induced apoptosis [38]. Also, in SiHa and HeLa tumor cells, DDP8 silencing by small interfering RNA (siRNA) inhibited proliferation, migration and invasion, in addition to inducing apoptosis [39]. All these data support our results obtained on MSCs when DPP8/9 activity is inhibited.

Substrates of DPP8/9 are still largely unknown. Nevertheless, it has recently been shown that the activity of these peptidases is involved in controlling the processing of more than 100 mitochondrial proteins, most notably adenylate kinase 2 (AK2). Therefore, DPP8/9 should play an important role in energy metabolism [40]. AK2 may have an intermembrane localization in mitochondria, regulating adenine-nucleotide interconversion. But it can also localize in the cytoplasm, where it is cleaved by DPP8/9 (predominantly, the latter), being therefore marked for proteosome degradation. In this way, DPP8/9 prevent an increase in cytoplasmic AK2, favoring mitochondrial localization [40]. If DPP8/9 activity is inhibited, AK2 accumulates in the cytoplasm. Then, it becomes active, inhibiting cell proliferation and inducing apoptosis through interaction with dual-specificity phosphatase 26 (DUSP26) [41]. Therefore, these data may partly explain our results, showing that DPP8/9 inhibition decreases viability, further increasing apoptosis of undifferentiated MSC induced to differentiate into osteoblasts. However, it is interesting to note that, in our study, adipocyte-induced MSC were not significantly affected in terms of viability when treated with a DPP8/9 inhibitor, as 1G244. This may be related to the fact that undifferentiated human MSC, and those differentiated to osteoblasts, maintain a high degree of proliferation. Yet, those differentiated into adipocytes lose division capacity when exposed to adipogenic inducers, in contrast to mouse 3T3-L1 preadipocytes [42]. Thus, results suggest that activation of apoptosis after DPP8/9 inhibition occurs mainly in proliferative and mitotically active cells.

In the case of osteoblastic differentiation of MSC, when cultures were treated with vildagliptin or 1G244, mineralization of the extracellular matrix was inhibited. This indicates that minimal levels of DPP8/9 activity are necessary for cell viability, as well as correct maturation of osteoblasts. Our results show that expression of genes encoding transcription factors RUNX2 and SP7 decreased with vildagliptin or 1G244 treatments. This was statistically significant in *RUNX2* expression, with inhibition of DPP8/9 by 1G244. Both transcription factors are critical for the initiation of osteoblastic differentiation. Therefore, if the expression of their encoding genes remains repressed during the differentiation process, osteoblastic differentiation does not occur [43,44]. Also, the use of 1G244 downregulated *IBSP* gene expression, whereas sitagliptin upregulated it. This is in line with the results obtained since the protein encoded by this gene is important in extracellular matrix formation in osteoblasts. Furthermore, *COL1A1* expression was repressed by 1G244. Collagen is the main bone-matrix protein and is essential for its mineralization [45]. Therefore, this result may explain the poor mineralization in these cultures.

Recently, it has been described that some drugs, like anagliptin, trelagliptin and saxagliptin, promote osteoblastic differentiation and mineralization of MC3T3-E1 mouse preosteoblastic cell line. This is accomplished through increased expression of *RUNX2* [46,47,48]. While anagliptin and trelagliptin are specific inhibitors of DPP4, saxagliptin also inhibits DPP8/9, as does vildagliptin [49]. Therefore, the results of the study with saxagliptin contradict what was observed in our case for vildagliptin. However, another study performed in vivo and in the same MC3T3-E1 cell type, as well as in rat-derived MSC with saxagliptin, concluded that, in rats, oral administration of this DPP4i alters long-bone microarchitecture. In the two cell types evaluated, saxagliptin treatment inhibited *RUNX2* expression and mineralization [50]. Saxagliptin concentrations used in the latter study were 1.5 and 15 µM. Both concentrations inhibited osteoblastogenesis, being in the range of that used in the study, where they observed the opposite effect. The best response on osteoblast differentiation was obtained with 2 µM of saxagliptin. Because both saxagliptin and vildagliptin have a certain ability to inhibit DPP8/9 [49], our results support that this type of DPP4i can negatively affect osteoblast differentiation and mineralization.

On the other hand, diabetes is a risk factor for osteoporosis [51]. Additionally, high plasma DPP4 levels in T2DM patients have been associated with bone loss and risk of fracture [21]. Thus, several studies have evaluated whether the use of DPP4i may impact bone health in humans. Currently, treatment with some DPP4i, like alogliptin, has been associated with decreased fracture risk. But in other cases, no such association has been reported [9,52]. In the case of vildagliptin, patients with T2DM treated for a year did not show any effect on bone metabolism [53]. The results of these studies have been mixed, so there are currently no conclusions on how DPP4i usage may affect bone metabolism. Moreover, recent meta-analyses suggest a neutral effect [54,55]. This supports our results obtained with sitagliptin, which did not significantly affect osteoblast mineralization. However, our data with respect to vildagliptin suggest that it may have negative effects on bone formation through inhibition of DPP8/9. However, it has been found that vildagliptin treatment improved trabecular-bone mineral density and microstructure in a model of obese, insulin-resistant, pre-diabetic rats [56]. Also, treatment with DPP4i promoted bone formation and reduced bone resorption by improving the microstructure of trabecular bones in a mouse model of T2DM [57]. These effects may be partly related to the improvement of glucose metabolism and glycemic control caused by DPP4i treatment, which may contribute to decreased bone loss [58].

With respect to adipogenesis, our results showed that the expression of *PPARG2*, *ATGL* and *LPL* genes was significantly repressed after 14 days with 1G244 treatment. The used cells are considered mature adipocytes at this stage. This may explain the decrease in lipid accumulation in these cultures. Therefore, these results suggest that DPP8/9 inhibition does not affect the initiation of adipogenesis but rather the maturation of adipocytes. It should be noted that inhibition of DPP4 by sitagliptin does not affect the differentiation of MSC into adipocytes. The 1G244 inhibitor has been shown to block adipogenesis in 3T3-L1 and 3T3-F422A preadipocytes, while DPP4 inhibitors had no effect. In addition, inhibition of DPP8/9 significantly affected the differentiation of preadipocytes into adipocytes. This highlights the relevance of DPP8/9 in adipogenesis, corroborating results obtained in this study [59].

However, the use of 1G244 does not allow us to determine which of the two—or if both—dipeptidyl peptidases (DPP8 or DPP9) is/are involved in adipogenic differentiation. Recently, however, a knockout mouse for DPP8 has been described as obese [60]. It is still unknown what role DPP8 may play in fat metabolism. Nevertheless, considering such data, our results suggest that DPP9 may have a relevant role in MSC differentiation into adipocytes. Indeed, although DPP8 and DPP9 present high homology between them, they do not share the same specificity for different substrate-cleavage sites. For example, DPP8 has lower specificity for Val-Ala sites than DPP9. Therefore, it has been suggested that each of these peptidases may have specific functions [61].

In adipocytes, the presence of DPP4 on the cell surface has been associated with a phenotype of high proliferation capacity and low differentiation potential. The opposite happened with DPP4^−^ preadipocytes [12,62]. However, soluble DPP4 synthesis increased during adipocyte differentiation, with adipocytes being one of the major sources of circulating DPP4 [63]. This indicates the different roles of DPP4 in adipogenesis. In our case, the use of specific DPP4i did not affect the adipogenic differentiation of MSC. This suggests that, as a whole, DPP4 activity is not determinant for MSC to reach adipocyte phenotype.

Among the functions that have been assigned to DPP8/9 are those of regulating energy metabolism. Specifically, the loss of DPP9 activity in DPP9 gene knock-in mice (DPP9 gki) produced important metabolic alterations in the liver and intestine of neonates [64]. Differentiation of MSC into osteoblasts and adipocytes leads to important metabolic changes. In the case of osteoblasts, there was a significant activation of the glycolysis pathway [65] and fatty-acid metabolism in adipocytes [66]. Therefore, inhibition of DPP8/9 could alter those metabolic changes, negatively affecting the differentiation process. Therefore, it would be interesting to study that in the future.

The Wnt/β-catenin signaling pathway plays an important role during the differentiation of osteoblasts and adipocytes [67]. In the case of osteoblastogenesis, activation of this pathway is necessary when MSC differentiate into preosteoblasts. Regarding adipocyte differentiation, the Wnt/β-catenin pathway must be inhibited [68]. Low-density lipoprotein (LDL) receptor-related proteins 5 and 6 (LRP5 and LRP6) participate in the activation of the canonical Wnt/β-catenin pathway. They are part of the LRP5/LRP6/Frizzled co-receptor group for Wnt proteins [69]. On the other hand, DKK1 is an antagonist of the Wnt/β-catenin signaling pathway that acts by sequestering LRP6 co-receptors. Thus, it cannot function together with frizzled receptors and thus activates the Wnt signaling cascade [70]. Therefore, the ratio of *DKK1*/(*LRP5* or *6*) expression is an indicator of the ability to activate or inhibit the Wnt/β-catenin pathway.

Our results show that, in osteoblasts, sitagliptin increased mRNA levels of the β-catenin encoding gene. However, it did not produce significant changes in protein synthesis. Similarly, vildagliptin did not affect the protein production of β-catenin. This is consistent with the fact that neither of the two DPP4i used produced changes in the *DKK1*/(*LRP5* or *6*) gene expression ratio with respect to untreated cultures. In the case of sitagliptin, this result was expected because, as our results showed, it did not produce significant changes in osteoblastic phenotype through extracellular matrix mineralization. Interestingly, sitagliptin decreased β-catenin expression under conditions of high glucose in the medium in other cell types, such as normal rat kidney (NRK)-52E, which are immortalized renal proximal-tubule epithelial cells [71]. On the other hand, sitagliptin is protected from apoptosis, partly through induction of the β-catenin pathway, in vascular smooth muscle cells (VSMC) exposed to oxidative stress with H_2_O_2_ [72]. This suggests that depending on cell type and culture conditions, this DPP4i may differently affect the Wnt/β-catenin pathway. This is probably related to the diversity of substrates that DPP4 can recognize.

In the case of vildagliptin, the lower degree of mineralization of vildagliptin-treated cultures cannot be explained by a decrease in β-catenin protein synthesis. Interestingly, 1G244 did inhibit β-catenin expression, at least in part, by inducing *DKK1* expression on day 14 of culture. Considering the role of the Wnt/β-catenin pathway on osteoblastogenesis, this correlates with the decrease in extracellular-matrix mineralization observed on day 21, when DPP8/9 activity was inhibited in osteoblast-induced MSC. Therefore, the possible effect of vildagliptin on DPP8/9 activity was not potent enough to repress β-catenin expression, suggesting the existence of another mechanism, explaining the negative effect of vildagliptin on mineralization.

In adipocytes, gene expression of *CTNNB1*, *DKK1*, *LRP5* and *LRP6* genes was unchanged on day 14 of differentiation, in sitagliptin- and vildagliptin-treated cultures, as compared to controls. However, β-catenin protein levels showed a tendency to decrease with these treatments, mainly with vildagliptin. Furthermore, as in osteoblasts, inhibition of DPP8/9 activity with 1G244 increased the *DKK1/*(*LRP5* or *6*) ratio and decreased the β-catenin protein. Inhibition of the Wnt/β-catenin pathway is important in the early stages of adipogenic differentiation. Nevertheless, it has been described that activation of the canonical Wnt/β-catenin pathway by different components is critical for adipocyte maturation and lipid metabolism in the final differentiation process [73]. In our study, by day 14 of differentiation, adipocytes were in their late maturation phase, with a significant accumulation of lipid droplets. Therefore, a decrease in Wnt/β-catenin pathway activity by vildagliptin or 1G244 may affect the maturation process of adipocytes. This may lead to a decrease in lipid-droplet formation, as we have shown by the oil red O staining of these cultures.

In conclusion, taken together, these results suggest that DPP8/9 activity, directly or indirectly, modulates the Wnt/β-catenin pathway in different physiological processes, including cell differentiation. Our results show that DPP8/9 activity plays an important role in maintaining the viability and differentiation of human MSC. This implies that non-specific DPP4i, such as vildagliptin, may have undesirable effects on MSC differentiation through its ability to partially inhibit DPP8/9. However, the effects produced by vildagliptin are not as potent as those generated by the specific DPP8/9 inhibitor, mainly in osteoblasts. This may explain why different studies have not observed significant clinical adverse effects in the use of this type of DPP4i [22]. Overall, our results suggest that specific DPP4i may have a higher degree of safety and that the use of potential inhibitors of DPP8/9 activity in human clinics should take into account their possible effects on MSC populations. Our results suggest that certain DPP8/9 substrates regulate the viability and differentiation of MSC. The accumulation of these substrates in cells, due to inhibition of DPP8/9 activity, must be partly responsible for the negative effects observed on viability and osteogenic and adipogenic differentiation of MSC. Therefore, in future studies, it will be important to identify these substrates to advance the knowledge of how DPP8/9 regulates these physiological processes of MSC. A limitation of this study is that it was performed in vitro. Thus, it would be interesting, taking into account our results, to study how the inhibition of DPP8/9 by non-specific inhibitors of DPP4 or by specific inhibitors of these enzymes can affect MSC populations at the level of viability and differentiation in preclinical models. This could provide more detailed information on possible repercussions that the clinical use of these inhibitors might have on the regenerative capacity of the organism and, in particular, on bone and fat metabolism.

## Figures and Tables

**Table 1:** Primer sequences and product sizes.

Gene	Primer Sequence (5′→3′)	Amplicon (bp)
Runt-related transcription factor 2 (*RUNX2*)	TGGTTAATCTCCGCAGGTCAC ACTGTGCTGAAGAGGCTGTTTG	143
Osterix (*SP7*)	AGCCAGAAGCTGTGAAACCTC AGCTGCAAGCTCTCCATAACC	163
Collagen, type I, alpha I (*COL1A1*)	CGCTGGCCCCAAAGGATCTCCTG GGGGTCCGGGAACACCTCGCTC	263
Integrin-binding sialoprotein (*IBSP*)	AGGGCAGTAGTGACTCATCCG CGTCCTCTCCATAGCCCAGTGTTG	171
Peroxisome proliferator-activated receptor gamma 2 (*PPARG2*)	GCGATTCCTTCACTGATACACTG GAGTGGGAGTGGTCTTCCATTAC	136
Patatin-like phospholipase domain containing 2 (*ATGL*)	CCAACACCAGCATCCAGTTCA ATCCCTGCTTGCACATCTCTC	102
Lipoprotein lipase (*LPL*)	AAGAAGCAGCAAAATGTACCTGAAG CCTGATTGGTATGGGTTTCACTC	113
Catenin beta 1 (*CTNNB1*)	AGCTGGTGGGCTGCAGAAAATG ACAATAGCCGGCTTATTACTAGAGC	249
Dickkopf1 (*DKK1*)	ATGCGTCACGCTATGTGCT GGAATACCCATCCAAGGTGCTA	144
LDL receptor-related protein 5 (*LRP5*)	TACTGGACAGACTGGCAGACC GTGTAGAAAGGCTCGCTTGG	209
LDL receptor-related protein 6 (*LRP6*)	TACTGGCCAAATGGACTGACT TGTTGCAAGCCAAAATGGAGT	211
Polymerase (RNA; DNA directed) II polypeptide A (*POLR2A*)	TTTTGGTGACGACTTGAACTGC CCATCTTGTCCACCACCTCTTC	125

## Data Availability

The data that support the findings of this study are available from the corresponding authors upon reasonable request.

## Supplementary Materials

The following supporting information can be downloaded at https://www.mdpi.com/article/10.3390/jcm12144632/s1: Figure S1. MSC cultures in passage 2 and at 80% confluence were raised with "Cell Dissociation Solution Non-enzymatic" (Sigma-Aldrich) and incubated with the anti-CD34-APC from Bio-Techne—Novus Biologicals (Minneapolis, MN, USA), anti-CD45-BB515 and anti-CD105-APC from Becton Dickinson Biosciences (BD; Franklin Lakes, NJ, USA), as well as anti-CD73-FITC and anti-CD90-PE-Cy5 (from eBioscience—Thermo Fisher Scientific) antibodies for 25 minutes in the dark. Cells were then washed with PBS (Sigma-Aldrich), 1% BSA from Tocris Bioscience (Bristol, UK) and 0.1% sodium azide (Sigma-Aldrich), and finally resuspended in CellFIX solution (Becton Dickinson Biosciences) before analysis on an LSRFortessa SORP flow cytometer from the same manufacturer. Results show that cells are positive for CD73, CD90 and CD105, but negative for CD34 and CD45 hematopoietic markers. Figure S2. Differentiation of MSC into adipocytes and osteoblasts. A: Light microscopy image of undifferentiated MSC, stained with oil-red O and hematoxylin. B: Same as in A, but MSC differentiated into adipocytes during 14 days. In this case, cells positive for oil-red O staining, specific for fatty vesicles, are observed. C: Well of P12 plate of undifferentiated MSC culture, stained with alizarin red. D: Same as in C. but MSC differentiated into osteoblasts for 21 days. Mineralization of extracellular matrix appears stained with alizarin-red S.
