# Supplementary material for: Non-Specific Inhibition of Dipeptidyl Peptidases 8/9 by Dipeptidyl Peptidase 4 Inhibitors Negatively Affects Mesenchymal Stem Cell Differentiation
Source: J Clin Med. 2023 Jul 12;12(14):4632. doi: 10.3390/jcm12144632 (PMC10380885; doi:10.3390/jcm12144632)
Supplement: Supplementary file 1 [file jcm-12-04632-s001.zip › jcm-2444389-supplementary.pdf]

Journal of
Clinical Medicine

SUPPLEMENTARY MATERIAL

Characterization of human bone-marrow MSC

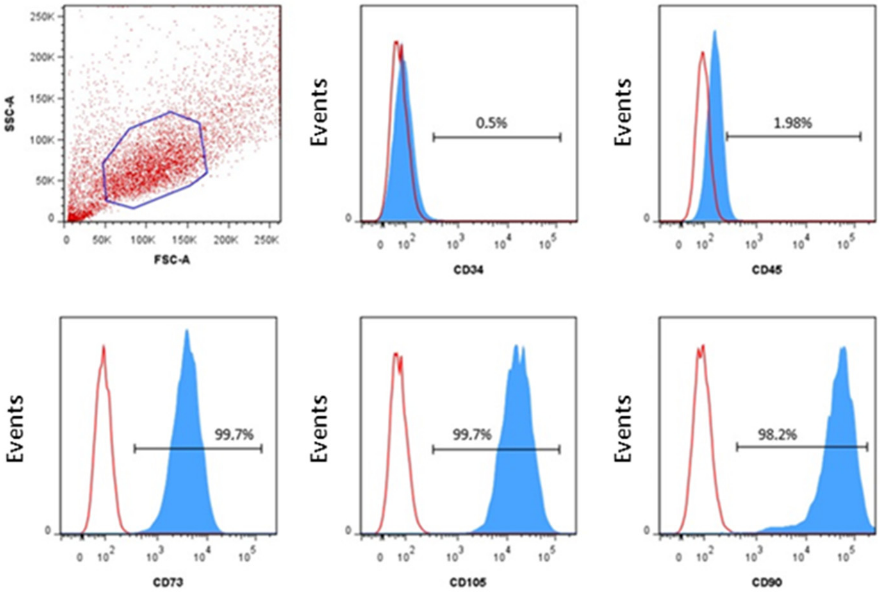

Figure S1. MSC cultures in passage 2 and at 80% confluence were raised with "Cell Dissociation Solution Non-enzymatic" (Sigma-Aldrich) and incubated with the anti-CD34-APC from Bio-Techne - Novus Biologicals (Minneapolis, MN, USA), anti-CD45-BB515 and anti-CD105-APC from Becton Dickinson Biosciences (BD; Franklin Lakes, NJ, USA), as well as anti-CD73-FITC and anti-CD90-PE-Cy5 (from eBioscience - Thermo Fisher Scientific) antibodies for 25 minutes in the dark. Cells were then washed with PBS (Sigma- Aldrich), 1% BSA from Tocris Bioscience (Bristol, UK) and 0.1% sodium azide (Sigma- Aldrich), and finally resuspended in CellFIX solution (Becton Dickinson Biosciences) before analysis on an LSRFortessa SORP flow cytometer from the same manufacturer. Results show that cells are positive for CD73, CD90 and CD105, but negative for CD34 and CD45 hematopoietic markers.

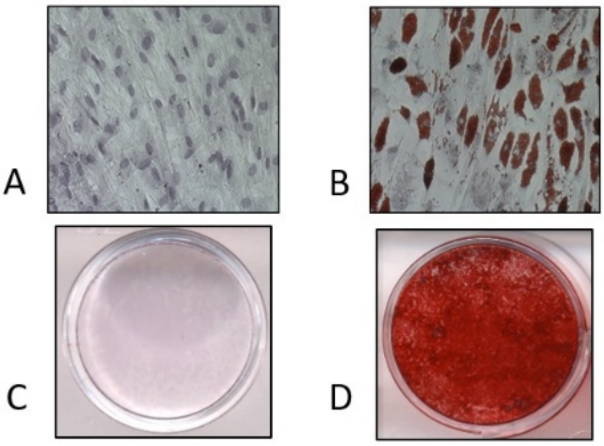

Figure S2. Differentiation of MSC into adipocytes and osteoblasts. A: Light microscopy image of undifferentiated MSC, stained with oil-red O and hematoxylin. B: Same as in A, but MSC differentiated into adipocytes during 14 days. In this case, cells positive for oil-red O staining, specific for fatty vesicles, are observed. C: Well of P12 plate of undifferentiated MSC culture, stained with alizarin red. D: Same as in C. but MSC differentiated into osteoblasts for 21 days. Mineralization of extracellular matrix appears stained with alizarin-red S.
